# Inferior Breast-Chest Contour Detection in 3-D Images of the Female Torso

**DOI:** 10.1109/JTEHM.2016.2614518

**Published:** 2016-09-29

**Authors:** Lijuan Zhao, Audrey Cheong, Gregory P. Reece, Michelle C. Fingeret, Shishir K. Shah, Fatima A. Merchant

**Affiliations:** 1 Department of Computer ScienceUniversity of Houston 14743 HoustonTX77204USA; 2 Department of Electrical and Computer EngineeringUniversity of Houston 14743 HoustonTX77204USA; 3 Department of Plastic SurgeryThe University of Texas MD Anderson Cancer Center 4002 HoustonTX77030USA; 4 Department of Behavioral ScienceThe University of Texas MD Anderson Cancer Center 4002 HoustonTX77030USA; 5 Departments of Engineering TechnologyElectrical and Computer Engineering, and Computer ScienceUniversity of Houston 14743 HoustonTX77204USA

**Keywords:** Breast morphology, 3D breast image, breast contour detection, lowest visible point, curvature analysis

## Abstract

Stereophotogrammetry is finding increased use in clinical breast surgery, both for breast reconstruction after oncological procedures and cosmetic augmentation and reduction. The ability to visualize and quantify morphological features of the breast facilitates pre-operative planning and post-operative outcome assessment. The contour outlining the lower half of the breast is important for the quantitative assessment of breast aesthetics. Based on this inferior breast contour, relevant morphological measures, such as breast symmetry, volume, and ptosis, can be determined. In this paper, we present an approach for automatically detecting the inferior contour of the breast in 3D images. Our approach employs surface curvature analysis and is able to detect the breast contour with high accuracy, achieving an average error of 1.64 mm and a dice coefficient in the range of 0.72–0.87 when compared with the manually annotated contour (ground truth). In addition, the detected contour is used to facilitate the detection of the lowest visible point on the breast, which is an important landmark for breast morphometric analysis.

## Graphical abstract

Detection of the inferior breast-chest (IBC) contour in 3D images of the female torso. First, we calculate the two principal curvatures and shape index for all points in the surface mesh of the 3D image. A set of possible contour points is determined next, consisting of points with negative shape indices (i.e., exhibiting concave shape) and a minimum principal curvature value less than the mean curvature of all points on the torso. The possible contour points include not only the points lying along the breast contour but also randomly scattered points in other regions on the torso that represent isolated incidences of low shape index and curvature values due to mesh undulation. A reference point, located on the breast mound roughly above the nipple position (the presence of a nipple is not required), is determined for each breast to facilitate separation of the breast contour points from the other points on the torso that also display low curvature values. Finally, cubic spline curve fitting is applied to the detected points, and the curve is identified as the breast contour. To find the lowest visible point for participants with ptosis grades of 1 or higher, the surface normal for each triangle of the surface mesh is determined, and triangles for which the surface normal is directed downward within 10 degrees of the z-axis are selected as potential points. This point set is then filtered to select only those points that lie between the first and last contour points of the selected breast. The lowest visible point is then determined to be the point with the lowest y-value within the set of points.

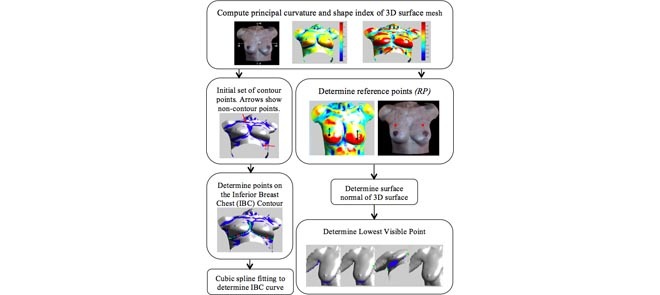

## I. Introduction

Stereophotography in conjunction with breast morphometry is now finding its niche in clinical breast surgery [1]–[9]. Three-dimensional (3D) surface images from stereophotography enable quantitative assessments of breast morphology, such as measurements of distances [1], breast volume [3], [4], breast ptosis [6], [7], and symmetry [8], [9]. Evaluating these different characteristics is essential for creating surgical plans that achieve aesthetically pleasing results and for assessing outcomes post-operatively.

Quantitative assessments of breast morphometrics typically involve the identification of key fiducial points, such as the sternal notch, nipples, and inframammary fold (IMF), that provide anatomical landmarks to delineate features of interest. The IMF, in particular, is an important landmark for assessing several objective measures of breast morphology. It is defined as the fold or crease that forms the lower border of the base of the breast and the chest wall. However, the visibility–and thereby detection–of the IMF is influenced by the shape of the breast, specifically sagging, or ptosis, of the breast [10], [11]. Ptosis occurs due to gravity acting on the breast, and increased ptosis is associated with pregnancy and breast feeding, not wearing a bra, and loss of elastic tissue due to aging.

**FIGURE 1.**
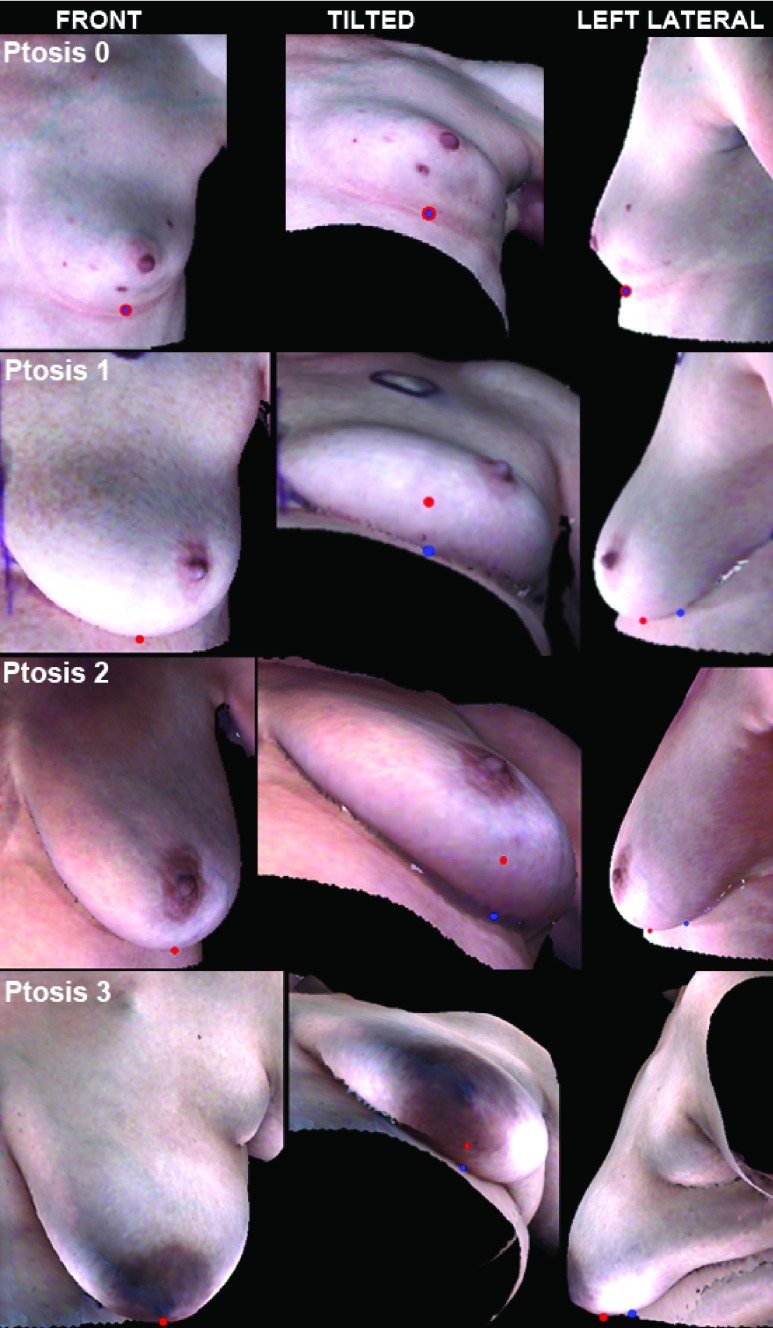
A point (blue) on the inferior breast-chest (IBC) contour and the lowest visible point (LVP) (red) are shown in 3 different views–front, tilted, and left lateral–for ptosis grades 0-3. In grade 0 ptosis (No Ptosis), the nipple and breast parenchyma (glandular tissue and fat which compose the breast) are located above the inframammary fold (IMF). In grade 1 ptosis, the nipple is at the level of the IMF and above the LVP. In grade 2 ptosis, the breast exhibits sagging in which the nipple lies below the level of the IMF but remains above the LVP of the breast, and in grade 3 ptosis, the breast exhibits severe sagging in which the nipple lies well below the IMF and lies at or below the LVP. Note that the total extent of IMF is only visible for a ptosis grade of 0 (i.e., IBC corresponds to the IMF) and is partially or completely obscured in other ptosis grades, and there is a difference in height between the LVP and IBC.

In order to overcome the limited visibility of the IMF in breasts of varying shapes, the terms lowest visible contour and lowest visible point (LVP) are used clinically to describe the lower border of the breasts. In women with breast ptosis, the lowest visible contour is the inferior-most contour of the breast that is visible with the woman in a standing position (and is typically much lower than the IMF), and the LVP is the inferior-most point along the lowest visible contour of the breast. In women without breast ptosis, the lowest visible contour and LVP are the same as the IMF. In this study we use the term inferior breast-chest (IBC) contour to represent the lowest breast contour in the 3D images, that is, the visible boundary along which the breast lies on the chest wall (see Fig. 1). Similar to the lowest visible contour, the IBC contour is the same as the IMF in women with no ptosis, and is much lower than the IMF in women with ptosis.

Manually annotating the lowest visible contour and the LVP, both in person and on images, is not only tedious and time consuming but also suffers from high inter- and intra-operator variability. In this novel study, we undertook the innovative use of shape and curvature for the detection of the inferior breast-chest contour directly from 3D images. To date, no algorithms that directly compute the lowest breast contour on 3D surface mesh images have been reported. Previous studies of breast contour detection have been limited to the detection of the contour outlining the lower half of the breast as it lays on the chest/abdominal wall and have used 2D photographs and/or 2D images encoding depth (depth-map images). Cardoso *et al.* [12], [13] described an automatic method for the detection of the lower half of the breast contour between an internal and an external end-point in 2D images. In subsequent studies, they detected the 2D outline of the lower half of the breast in photographs and range images [14], [15].

Lee *et al.* [16], [17] introduced a measure of the lower half of the breast contour in 2D images, which enforced a mathematical shape constraint based on the catenary curve, a perfectly flexible and inextensible string of uniform density supported by 2 distinct points. The catenary-based shape measure was used by Lee *et al.* to evaluate the contours of the upper and lower breast in 3D images of patients [18] and breast ptosis in 2D images [19]. Although this method used 3D images as input, the obtained breast contours were curves in 2D planes and did not directly mirror the 3D breast contours.

We describe a curvature-based IBC contour detection algorithm in 3D images of the female torso that employs the shape index and minimum principal curvature [20]. As evidenced by the published work of Cardoso *et al.* [12], [13], Oliveira *et al.* [14], [15], and Lee *et al.* [16]–[19], the detection of the breast contour has several practical applications such as for the aesthetic evaluation of breast cancer treatment [14] and the detection of prominent points on the torso [15], breast curvature [16], [18], and ptosis.

The IBC contour is important for determining symmetry between the left and right breasts, especially as it relates to how symmetrical the breasts are as suspended from the chest wall. Therefore, in this study, we present a robust algorithm to automatically detect the IBC contour in breasts of all shapes and sizes. The IBC contour can be used to (1) create a contour analysis to profile the shape of the lower pole of the breast, (2) identify the IMF in breasts without ptosis, (3) assess symmetry, and (4) perform automated segmentation of the breast mound from images. We also demonstrate the utility of our IBC contour detection algorithm for enabling the detection of the LVP, which is used as a landmark by clinicians to assess ptosis and breast symmetry but difficult to annotate manually.

## II. Methods

**FIGURE 2.**
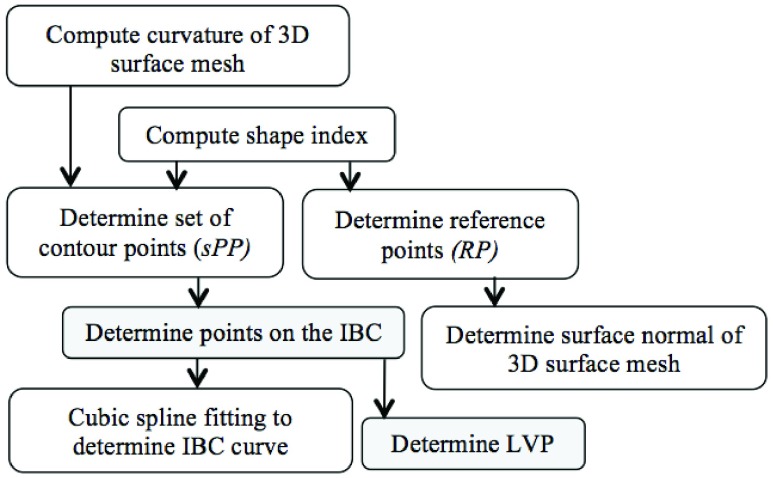
Flowchart of the algorithm for the detection of the inferior breast-chest (IBC) contour in 3D images of the female torso.

Female patients undergoing breast reconstruction surgery at The University of Texas MD Anderson Cancer Center and commissioned volunteers were recruited under protocols approved by the institutional review board. All participants enrolled in the study received $20 per study visit. Three-dimensional images were obtained using the 3dMDTorso system (3dMD LLC, Atlanta, GA). For contour detection, the region of interest (ROI) was defined as the region starting below the neck and extending to just above the umbilicus. The ROI was manually cropped using customized software [1]. A flowchart of the algorithm for the detection of the IBC contour in 3D images is illustrated in Fig. 2.

First, we calculate the 2 principal curvatures for all points in the surface mesh of the 3D image. Then the shape index is determined from the 2 principal curvatures [20]. A set of possible contour points ($sPP)$ is determined next, consisting of points with negative shape indices (i.e., exhibiting concave shape) and a minimum principal curvature value less than the mean curvature of all points on the torso. The possible contour points include not only the points lying along the breast contour but also randomly scattered points in other regions on the torso that represent isolated incidences of low shape index and curvature values due to mesh undulation. A reference point $RP$, located on the breast mound roughly above the nipple position (the presence of a nipple is not required for determination of the $RP)$, is determined for each breast to facilitate separation of the breast contour points from the other points on the torso that also display low curvature values. Finally, cubic spline curve fitting is applied to the detected points, and the curve is identified as the breast contour.

### A. Curvature Analysis

Curvature is defined as the amount that a surface deviates from being flat. At each vertex point $p$ of a 3D triangular surface mesh, one may find a normal plane, which contains the normal vector of the point $p$. The intersection of the normal plane and the 3D surface is a plane curve. The plane curves from different normal planes at point $p$ will generate different curvatures. The principal curvatures, $k_{max}$ and $k_{min}$, are the maximum and minimum values of the curvatures at $p$. To calculate the principal curvatures on the 3D surface mesh, we used a toolbox developed by Peyre [21] based on the algorithms proposed by Cohen-Steiner and Jean-Marie [22] and Alliez *et al.* [23].

### B. Shape Index

The shape index $S$ for each point on the surface mesh was computed using the formula proposed by Cantzler and Fisher [20]:

\begin{equation*} S= \frac {2}{\pi } {tan}^{-1}\left ({ \frac {k_{max}+k_{min}}{k_{max}-k_{min}} }\right ) \end{equation*}

**FIGURE 3.**
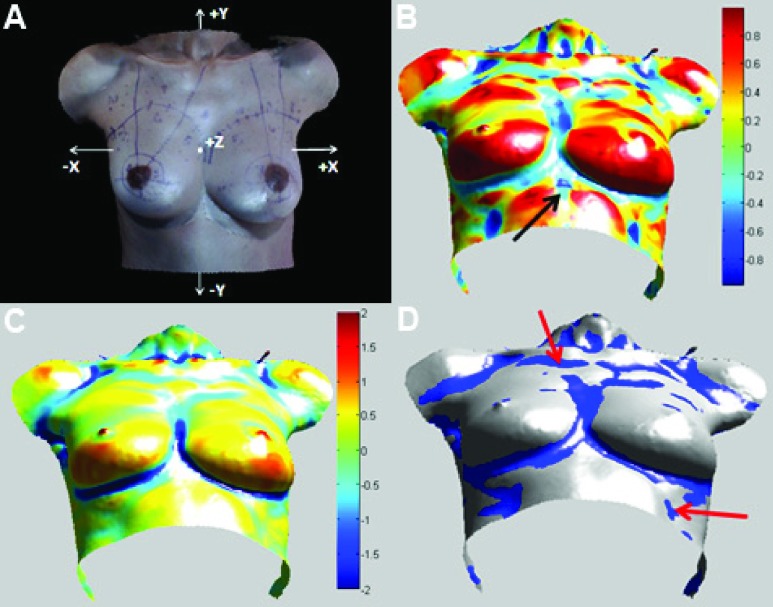
(A) Representative ROI from a 3D image of the female torso ($X$, $Y$, and $Z$ axes are displayed in the figure). (B) Color-mapped shape index of 3D surface mesh. The black arrow illustrates the region eliminated from $sPP$ by $k_{min}<k_{mean}$. (C) Color-mapped minimum principal curvature $k_{min}$ of 3D surface mesh. The curvature values are centered so that the $k_{mean}$ is 0 (green), i.e., the values above $k_{mean}$ are shown to be positive (yellow to red, convex) and the values below $k_{mean}$ are shown to be negative (light blue to dark blue, concave). (D) Regions of possible contour points $sPP$ (blue) in the surface scan. Red arrows illustrate points on the torso that exhibit low curvature and thus are in the $sPP$ set but do not lie along the breast contour.

We employ a pseudo-color visualization method for viewing the shape index of the 3D mesh. Fig. 3(a) presents a representative 3D image of the torso, and the color-mapped shape index for the torso is presented in Fig. 3(b). The region of the lower breast mound is red ($S>0$, convex shape) and the region of the breast contour is blue ($S<0$, concave shape).

### C. Set of Candidate Contour Points

Following curvature and shape index computation, we first obtain a set of possible contour points, $sPP$, to contain all points in the ROI that have a negative $S$ value and a minimum principal curvature $(k_{min})$ less than the mean of the minimum principal curvatures $(k_{mean})$ for all points in the ROI. For a point $p$ in the ROI:

\begin{equation*} p\in sPP~if~S<0~\And ~k_{min}<k_{mean} \end{equation*}

Points in regions that are relatively flat exhibit $k_{max}$ and $k_{min}$ values close to zero. However, in these regions $ k_{max}+k_{min}<0$ may be met and, thereby, $S<0$. We use the condition $k_{min}<k_{mean}$ to eliminate these points from the $sPP$. In Fig. 3(b), the region indicated by the black arrow contains points that meet the condition $S<0$ but fail the condition $k_{min}<k_{mean}$. Thus, the combination of $S<0$ and $k_{min}<k_{mean}$ filters the set $sPP$ such that it has few points that have low curvatures and are not along the breast contour.

Fig. 3(c) shows the color-mapped minimum principal curvature $k_{min}$ of the 3D surface mesh. In the figure, we center the minimum principal curvatures so that $k_{mean}$ is 0, i.e., the values above $k_{mean}$ are shown to be positive (yellow to red) and the values below $k_{mean}$ are shown to be negative (light blue to dark blue). The set of possible contour points ($sPP)$ is displayed in blue in Fig. 3(d).

### D. Determination of Reference Point for Each Breast Mound

**FIGURE 4.**
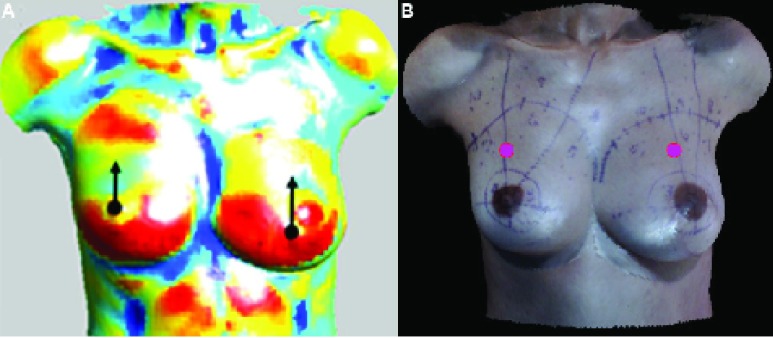
(A) Color-mapped average of the weighted shape index ($ave_{S}$) in $5mm\times 5mm$ blocks. The black dot indicates the position of block A, and the arrow indicates the search range ($7 \times 10$ blocks) above block A, within which block B is detected as the block with the largest $ave_{S}$ in the vertical direction. (B) Estimates of reference points $(RPs $, magenta) for the left and right breasts.

Following the determination of the possible contour points set $sPP$, we automatically locate an estimate for the reference point $RP$ (see Fig. 4) for each breast using the shape index.

$RP$ is used as a reference point to calculate angles with respect to the Y-axis for points in set $sPP$. The algorithm for the determination of the $RP$ leverages the anatomical shape of the breast [10], wherein the inferior pole exhibits largely convex contouring. The $RP$ is thus determined to be the point that lies above the area where the contouring of the lower pole just begins to slope downward toward the chest wall (indicated in Fig. 4(a) by the black dot at the start of the black arrow). The $RP$ determination method is applicable to both breasts, but for simplicity, it is discussed here in terms of the right breast only. To determine the $RP$, we use the weighted shape index for each point, where the weight is the $z$ coordinate value of the point. Initially, the points on the right half of the ROI are divided into blocks based on their $x$ and $y$ coordinates ($x$, $y$, and $z$ directions are displayed in Fig. 3(a)), and the average of the weighted shape index, $ave_{S}$, is computed for each block of an empirically determined size of $5mm \times 5mm$. The 3D point cloud is dense in the breast mound region, with typical distances between adjacent points in this region at $\leq 2mm$. A $5mm \times 5mm$ block includes approximately 9 points and allows computation of a smoothed weighted shape index for the points. For each block $i$, the average of the weighted shape index, ${ave_{S}}_{i}$, is computed as follows:

\begin{equation*} {ave_{S}}_{i}= \frac {\sum _{j=1}^{n_{i}} {S_{ij}z_{ij}}}{n_{i}}, \end{equation*}

where $n_{i}$ is the number of points in block $i$, and $S_{ij}$ and $z_{ij}$ are the shape index and $z$ coordinate of point $j$ in block $i$, respectively. $z_{ij}$ is normalized so that all points on the right half of the torso have non-negative $z$ coordinate values:

\begin{equation*} {z_{ij}=zo}_{ij}-z_{min}, \end{equation*}

where ${zo}_{ij}$ is the original $z$ coordinate value of point $j$ in block $i$, and $z_{min}$ is the minimum original $z$ coordinate value for all points on the right half of the torso. Fig. 4(a) shows the color mapped $ave_{S}$ for each $5mm \times 5mm$ block of the torso. In the right half of the torso, the block with the largest $ave_{S}$, block $A$, is shown as a black dot (see Fig. 4(a)). Based on anthropometric measurements of breast morphology [24], a range of $7\times 10$ blocks ($35mm \times 50mm$, i.e., $15mm$ from block $A $in the left and right directions) above this block is examined, and the highest block (in the $y$ direction) with an $ave_{S} > 0$ is designated as block $B$. The coordinates of the $RP$ were estimated as follows (only $x$ and $y $coordinates of $RP$ are required for angle calculation for possible contour points in set $sPP)$:

\begin{equation*} \begin{cases} x_{RP}=x_{AC}\\ y_{RP}=y_{BC,}\\ \end{cases} \end{equation*}

where $x_{RP}$ and $y_{RP}$ are $x$ and $y$ coordinates of $RP$, respectively, $x_{AC}$ is the $x$ coordinate of the center of block $A$, and $y_{BC}$ is the $y$ coordinate of the center of block $B$. The $15mm$ from block $A$ in the left and right directions is used to ensure that the $RP$ is not far from block $A$ in the $x$ direction. A distance of $50mm$ above $A$ is used to avoid an $RP$ location lower than the breast contour in images of ptotic breasts. The automatically estimated $RP$ locations for 2 breasts are shown in magenta in Fig. 4(b).

### E. Determination of the Inferior Breast-Chest Contour

#### 1) Angle Calculation

For each point $p_{i}$ in the possible contour points set $sPP$ from the right half of the torso, we calculate the angle $\theta _{i}$, which is relative to $RP$ and defined by:

\begin{equation*} \theta _{i}= {sign(x_{p_{i}}-x_{RP})cos}^{-1}\left ({ \frac {\vec {v_{1}}\cdot \vec {v_{2}}}{\vert \vec {v_{1}\vert }\cdot \vert \vec {v_{2}\vert }} }\right ), \end{equation*}

where $\vec {v_{1}}$ is a vector along the $-y$ direction, $\vec {v_{2}}=(x_{p_{i}}-x_{RP}, { y}_{p_{i}}-y_{RP})$ in which $x_{p_{i}}$ and $y_{p_{i}}$ are $x$ and $y$ coordinates of point $p_{i}$ in $sPP$, and $x_{RP}$ and $y_{RP}$ are coordinates of $RP$. All points in set $sPP$ are sorted based on their angles to facilitate subsequent computations.

#### 2) Intermediate Point Determination

We divide points in the $sPP$ into different sectors based on their angles and detect 1 breast contour point in each sector. The normal breast base width is no more than $20cm$ [2], [24]. We divide points in the $sPP$ into different sectors at an angle interval of 5°; then, the average distance between 2 adjacent detected breast contour points is no more than $13mm$. This point density is high enough to fit a contour curve using cubic spline [25]. A smaller angle interval can be selected, however, with a trade-off of longer computation time.

**FIGURE 5.**
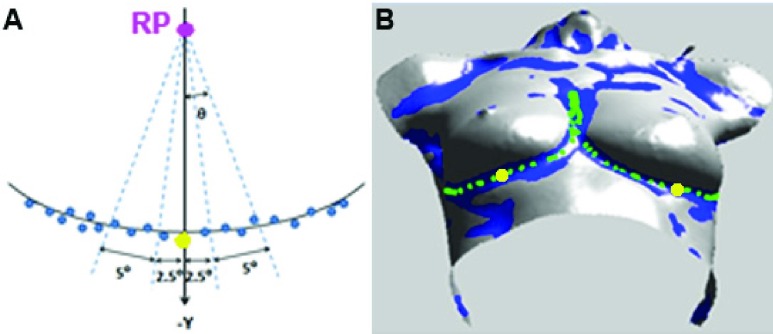
(A) $sPP$ points (blue) are divided into different 5° sector regions based on their angles $\theta $ relative to the reference point ($RP$, magenta). The yellow point is point $M$, the possible contour point displaying the largest shape change. (B) Set of possible contour points $sPP$ (blue) and detected points along the breast contours (green) displayed on the surface. Yellow points are the determined intermediate points.

From the sector below the $RP$, i.e., −2.5° ~ 2.5° in the $sPP$ (Fig. 5 (a)), we estimate an intermediate point of the breast contour, which is used to locate the contour position correctly. Points in this sector have $x$ coordinates close to that of the $RP$. The intermediate point is determined using the following 3 steps: (1) calculate normals for all points in sector −2.5° ~ 2.5° in the $sPP$; (2) find the possible contour point $M$ displaying the largest shape change; and (3) estimate the intermediate point from $M$.

For each point in the sector −2.5° ~ 2.5° in the $sPP$, the normal is calculated as the sum of the normalized normals of its one-ring triangles. One-ring triangles of a point $p$ in the triangular surface mesh are defined as all the triangles that share point $p$.

The possible contour points in set $sPP$ include not only the points in the region containing the breast contour but also randomly scattered points in other regions in the ROI that represent isolated incidences of low shape index and curvature values due to mesh undulation (Fig. 3(d)). However, below the $RP$, only the breast contour area exhibits a sharp change in shape (Fig. 5(b)). In the sector −2.5° ~ 2.5°, we find a possible contour point $M$ displaying the largest shape change in a range with radius $10mm$ around the point to locate the region containing the breast contour. Before arriving at a radius size of 10 mm, we evaluated a range of different radii: $5mm$, $10mm$, $15mm$, and $20mm$. Since the point cloud is sparse in the breast contour region for some 3D images, the distance between some adjacent points may be larger than $5mm$. That is, a radius of $5mm$ was empirically determined to be inadequate. Similarly, with a radius of $20mm$, the detected intermediate point may be located outside of the breast contour region, making this radius too large. Radii of $10mm$ and $15mm$ both obtain accurate intermediate point estimation, and we selected $10mm$ as the radius size.

The point $M$ (yellow in Fig. 5(a)) is estimated by angles between the normals of points, i.e., the larger the angle between the normal vectors of 2 points, the larger the shape changes between them. For each point $p$ in the sector −2.5° ~ 2.5° in the $sPP$, we calculate the angles of normals between point $p$ and each of the other possible contour points within a range of $10mm$ in Euclidean distance to $p$ and let the maximum angle be the normal angle ($NOA)$ of $p$. The point $M$, with the maximum $NOA$ in the sector −2.5° ~ 2.5° is selected as the possible contour point.

The intermediate point (Fig. 5(b)) is selected based on the observation that the breast contour is an inward curving crease below the breast and the points on the contour exhibit low minimum principal curvatures. We determine the intermediate point as the point in the $sPP$ that is in a range of $10mm$ in Euclidean distance to point $M$ and has the minimum $k_{min}$ value.

#### 3) Curvature Extension

From the estimated intermediate point of the IBC contour, we extend the contour points along 2 directions. $sPP$ points are divided into different 5° sector regions centered at $RP$ (Fig. 5(a)). In each sector, we detect the contour point, such that it has a minimum $k_{min}$ value in all $sPP$ points in this sector and the Euclidean distance to the detected contour point in the previous interval is $<2L$. $L$ is the arc length of the current interval and can be calculated as shown below:

\begin{equation*} L=\frac {5^{o}\pi }{180^{o}}R, \end{equation*}

where $R$ is the radius from $RP$ to the contour arc of the current interval, which is approximated as the Euclidean distance from $RP$ to the detected contour point in the previous interval (since the current interval has not yet undergone processing to separate the breast contour point from noise). The distance 2L is the largest possible distance between the breast contours in the adjacent sectors. It is used to avoid selecting contour points outside the breast contour region. If there is no $sPP$ point in an interval at distance $<2L$, the detection is terminated in that direction. The detected contour points are displayed in Fig. 5(b) in green. The resulting fitted cubic spline curve [25] generated from the detected contour points is identified as the IBC. It should be noted that this is the curve where the breast lies on the chest wall in the 3D images and not the curve of the breast mound on which the lowest visible point is located.

### F. Determination of the Lowest Visible Point

**FIGURE 6.**
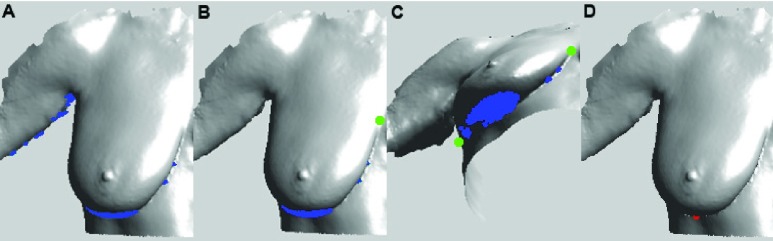
Detection of the lowest visible point (LVP). (A) Points (blue) on the 3D mesh for which the surface normal is directed downward within 10 degrees of the z-axis. (B-C) Front and tilted view showing the filtered set of points that lie between the first and last contour points (shown in green) of the breast. (D) LVP (red) determined as the point with the lowest y-value.

Next, to find the lowest visible point for participants with ptosis grades of 1 or higher, we computed the surface normal for each triangle of the surface mesh as follows. For each triangular face of the mesh, if $a$ and $b$ are the 2 vectors denoting the 2 sides, then the normal vector is defined as $normal=a\times b$. Triangles for which the surface normal is directed downward within 10 degrees of the z-axis are selected as potential points (Fig. 6(a)). This point set is then filtered to select only those points that lie between the first and last contour points of the selected breast (Fig. 6(b-c)). The lowest visible point is then determined to be the point with the lowest y-value within the set of points (Fig. 6(d)).

## III. Evaluation Metrics

We demonstrate our proposed breast contour detection algorithm for 3D images by comparing the automatically detected contours with manually selected contours. Using customized software [1], 1 member of our group (LZ) manually selected points along the breast contour on the 3D surface images. The manually selected contours were used as ground truth. The average distance between the automatically detected and manually selected contours from the same breast and the dice coefficient were computed for comparison.

### 1) Average Distance

The average distance between 2 IBC contours is the average of the distances between all points in 1 of the contours and the corresponding points in the other contour, as discussed below. The automatically detected contour and manually selected contour from the same breast are unequal in length. To evaluate the accuracy of our proposed algorithm, we normalized the lengths for comparison as follows.

The cubic spline approach [25] was used to obtain 2 interpolated point sets, $A$ and $B$, for the automatically detected and manually selected contour points, respectively, such that each set has an equal number of points with similar length. A total of 200 points were interpolated in each contour for evaluation. The distance $d\left ({ A_{i} }\right )$ from a point $A_{i}$ in set $A$ to the other contour, i.e., set $B$, can be represented as:

\begin{equation*} d\left ({ A_{i} }\right )=\min _{{B_{j}}\in B}\left \|{ A_{i}-B_{j} }\right \|, \end{equation*}

where $\left \|{ \cdot }\right \|$ is the Euclidean distance. Similarly, the distance $d\left ({ B_{i} }\right )$ from a point $B_{i}$ in set $B$ to set $A$ can be represented as:

\begin{equation*} d\left ({ B_{j} }\right )=\min _{{B_{j}}\in B}\left \|{ B_{j}-A_{i} }\right \| \end{equation*}

Then the average distance $ave_{d}$ between the automatically detected contour and the manually annotated contour is calculated as follows:

\begin{equation*} ave_{d}=\frac {\sum _{i=1}^{\left |{ A }\right |} {d\left ({ A_{i} }\right )+\sum _{j=1}^{\left |{ B }\right |} {d\left ({ B_{j} }\right )}}}{\left |{ A }\right |+\left |{ B }\right |}, \end{equation*}

where $\vert A\vert $ and $\vert B\vert $ are sizes of the set $A$ and $B$, respectively.

### 2) Dice Coefficient

We computed the dice coefficient [26], which is a similarity measure, to compare the automatically detected breast contour and the manually annotated contour as follows. For each point in $A$ (or $B)$, we compute the distance to the other contour point set $B$ (or $A)$. Let $num$ be the total number of the points in $A$ and $B$ with distances less than a given threshold for comparison. The dice coefficient $Dc$ is computed as$ num$ over the sum of the total number of points in $A$ and $B$:

\begin{equation*} Dc=\frac {num}{\left |{ A }\right |+\vert B\vert } \end{equation*}

The dice coefficient is always in the [0, 1] range. A dice coefficient of 1 indicates high similarity (all points in $A$ and $B$ fall within a given distance threshold), whereas 0 indicates little to no similarity (all points in $A$ and $B$ fall outside the given distance threshold).

## IV. Dataset

A total of 77 3D surface images from 39 breast cancer patients and 5 volunteers were used in this study. Both breasts were included in all 77 images. From the 77 surface images, a total of 151 breast contours were automatically detected, while 3 breasts contours could not be detected due to the presence of holes in the surface mesh or missing data in the images. The 3D images were acquired at a single time point from volunteers, whereas for patients, images were longitudinally acquired during multiple visits as they underwent breast reconstructive surgery. The 3D images from 4 different visits were included for 1 participant, from 3 different visits for 9 participants, from 2 different visits for 12 participants, and from a single visit for 22 participants. The study population was 93.2% white, 2.3% African American, and 2% other. In terms of ethnicity, 90.9% were not Hispanic/Latino and 9.1% were Hispanic/Latino. Based on body mass index (BMI), 36% were normal (BMI = 18.5 ~ 24.9, 35% were overweight (BMI = 25 ~ 29.9, and 29% were obese (BMI ≥ 30). The age range was from 21 to 66 years.

To assess whether the proposed algorithm is able to detect the breast contour irrespective of the presence or absence of a nipple or the shape and size of the breast, we categorized our dataset based on ptosis grade (as defined in Fig. 1) and the presence of nipples.

The dataset was partitioned based on images of breasts with nipples ($N_{p}=90)$ and without nipples ($N_{a}=61)$. The 90 breasts with nipples were assigned a ptosis grade by 1 member of our group (GPR) and then categorized into 4 groups according to the degree of ptosis as follows: 46 breasts with grade 0 ptosis, 15 breasts with grade 1 ptosis, 16 breasts with grade 2 ptosis, and 13 breasts with grade 3 ptosis. The group of 61 breasts without nipples was not rated (NR) for ptosis.

## V. Results

**FIGURE 7.**
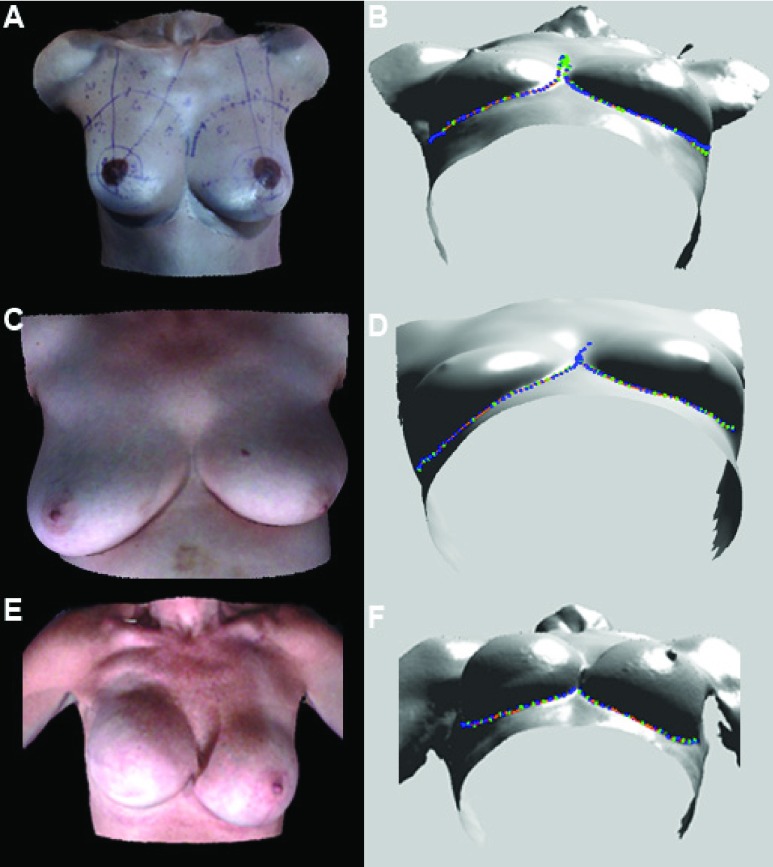
IBC detection results for 3 participants (A, C, E). The detected inferior breast-chest (IBC) contour points are shown in green with the estimated cubic spline curve in orange, and manually annotated points are in blue (B, D, F). (A) 3D image wherein both breasts have a ptosis grade 0. Left breast was reconstructed using a TRAM flap. (C) 3D image wherein the native right breast has a ptosis grade 2 and the left breast has a ptosis grade 3 after a segmental mastectomy. (E) 3D image wherein the right breast is undergoing breast reconstruction with an implant. This breast cannot be rated for ptosis since the nipple has not yet been reconstructed. The left breast has ptosis grade 1 after a mastopexy and breast augmentation.

We evaluated our proposed IBC contour detection algorithm using the 77 3D surface images for a total of 151 breasts. A subset of 5 surface images, i.e. a total of 10 breasts, was used for algorithm development, and testing was performed on the entire dataset. Data for 3 representative participants are presented in Fig. 7.

The IBC contour detection results are displayed in Fig. 7(b), (d), and (f). Blue points are the manually selected contour points, which were used as ground truth for comparison. Green points are the breast contour points detected using our proposed algorithm. The orange curve was obtained via cubic spline fitting of the detected contour points in green. As seen in Fig. 7(b), (d), and (f), high correspondence was achieved between the manually selected points and the automatically detected breast contours. Table I presents the mean of the average distances (i.e. detection error) and dice coefficients for automatically detected versus manually annotated breast contours for the 151 breasts analyzed.

**TABLE I:** Mean Contour Detection Errors for Total 151 Breasts

Average distance ($mm$)	Dice coefficients by distance thresholds					
	$0.5mm$	$1mm^{}$	$2mm^{}$	$3mm^{}$	$4mm^{}$	$5mm^{}$
1.64±0.76	0.21±0.11	0.41± 0.15	0.72± 0.16	0.87± 0.12	0.94± 0.07	0.97± 0.05

The mean detection error between automatically detected and manually annotated IBC contours for all distance thresholds was $1.64\pm 0.76 mm$. The mean detection error is less than 2 mm and falls within the acceptable range for clinical application.

We tested dice coefficients using 6 separation distance thresholds: $0.5mm$, $1.0mm$, $2.0mm$, $3.0mm$, $4.0mm$, and $5.0mm$. The distance threshold for dice coefficients represents the separation between the points on the 2 contours. The mean of the dice coefficients is the average value for the 151 breasts at a given separation distance threshold. From Table 1 we can see that as the distance threshold for similarity between 2 contours increased, the dice coefficient also increased. At a separation distance in the range of $4mm-5mm$ between the automatically detected and manually annotated IBC contours, we had very high dice coefficient values (0.94–0.97). At a resolution of $2mm-3mm$ the similarity was 0.72 – 0.87, and it was reduced only for very low threshold values of $1mm$ (0.41) and $0.5mm$ (0.21).

Table 2 shows the mean of average distances between detected IBC contours and ground truth contour points for the 4 ptosis grades and the breasts with no ptosis rating (NR) for the 151 breasts analyzed. The means of average distances for breasts in these 5 groups ranged from $1.29\pm 0.28mm~{\textrm {to}}~ 1.73\pm 0.98mm$. The minimum average distance error occurred for breasts with major ptosis (grade 3; $1.29\pm 0.28mm)$.

**TABLE II:** Mean of Average Distances$(mm)$ by ptosis grade.

Grade 0 (46 breasts)	Grade 1 (15 breasts)	Grade 2 (16 breasts)	Grade 3 (13 breasts)	NR (61 breasts)
1.73± 0.98	1.40± 0.43	1.62± 1.09	1.29± 0.28	1.72± 0.55

NR: Cannot be rated.

Table 3 shows the mean of the dice coefficients for the different distance thresholds for the 5 groups. For the $2mm-3mm$ distance threshold, the dice coefficient values for the 5 groups were 0.67 – 0.93; for the threshold range of $4mm-5mm$, the dice coefficients were 0.93 – 0.99. At a low separation distance of $0.5mm - 1mm$, the dice coefficient values were 0.18 – 0.47.

**TABLE III:** Mean Dice Coefficients for Five Ptosis Groups by Distance Threshold

Group	Distance threshold					
	$0.5mm$	$1mm^{}$	$2mm^{}$	$3mm^{}$	$4mm^{}$	$5mm^{}$
Grade 0 (46 breasts)	0.23± 0.11	0.42± 0.15	0.72± 0.14	0.88± 0.11	0.94± 0.07	0.97± 0.05
Grade 1 (15 breasts)	0.22± 0.12	0.45± 0.15	0.77± 0.13	0.90± 0.10	0.96± 0.06	0.99± 0.02
Grade 2 (16 breasts)	0.22± 0.13	0.45± 0.20	0.74± 0.20	0.89± 0.13	0.95± 0.09	0.97± 0.08
Grade 3 (13 breasts)	0.21± 0.06	0.47± 0.10	0.82± 0.09	0.93± 0.07	0.97± 0.05	0.98± 0.04
NR (61 breasts)	0.18± 0.10	0.36± 0.14	0.67± 0.17	0.84± 0.13	0.93± 0.08	0.97± 0.05

NR: Cannot be rated.

The best and worst agreement between the automatically detected and manually annotated IBC contours was noted for breasts exhibiting major and no ptosis, respectively, while for breasts not rated (NR) for ptosis, the agreement was moderate. This finding is reasonable in that our algorithm is founded on curvature and shape measurements and, thus, can precisely identify the prominent sagging along the lower breast pole that is observed in grade 3 ptosis but is less adept at identifying the breast contour in breasts with no ptosis wherein sagging is absent. Likewise, non-rated breasts include ptosis grades ranging from 0 to 3, and thus performance for this group was moderate.

**FIGURE 8.**
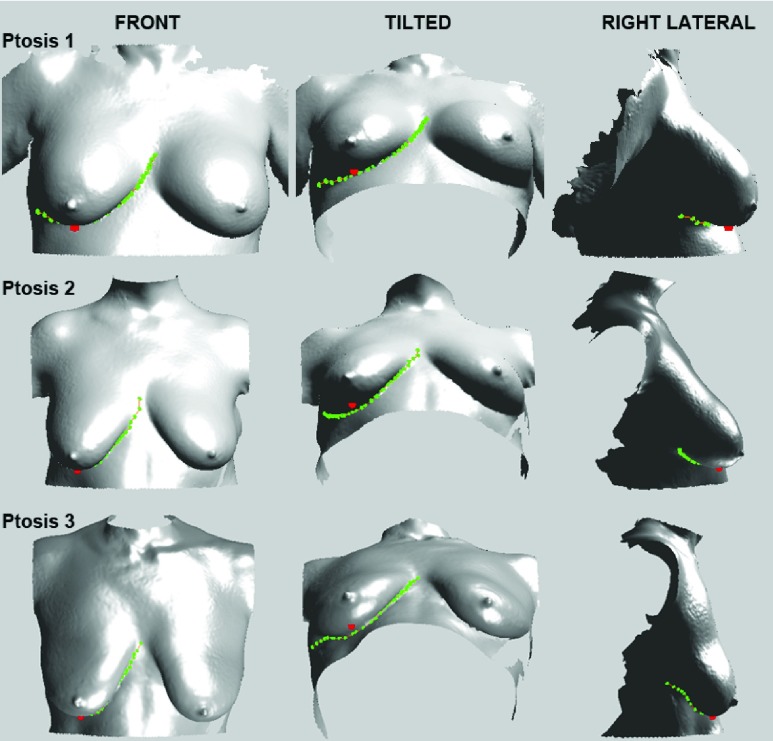
The detected lowest visible point (LVP; red), inferior breast-chest (IBC) contour points (green), and estimated cubic spline (orange) for participants with ptosis grades 1, 2, and 3, visualized on the right breast in the front, tilted, and right lateral views.

Following the detection of the IBC contour, the LVP was detected and superimposed on the 3D surface images for visualization. The rationale was to facilitate the visualization of the LVP on the breast, since plastic surgeons typically use the nipple in relationship to the IMF and this point as a secondary landmark for grading ptosis. Figure 8 presents representative images showing the detected LVP in participants with ptosis grades 1-3.

## VI. Conclusion

We have developed a curvature-based IBC contour detection algorithm for 3D images of the female torso. The algorithm uses established measures of curvature in conjunction with anthropometric knowledge of breast anatomy to automatically detect the inferior contour of the breast. The algorithm uses empirically determined values for the bounding box in reference point determination and the sector angle range for the intermediate point detection. These empirical values are based on established anthropometric measures of the female breast [10], [24].

Collectively, the results in this study validate the robustness of the proposed algorithm for the automated detection of the IBC. Most importantly, the algorithm proposed can be generalized to 3D images from any patient irrespective of their race and ethnicity. This is because the proposed algorithm is founded on identification of surface curvature and shape, both of which are independent of race and ethnicity, in contrast to skin color and texture. Although, variations in skin color and texture can be large across the different racial groups, the ranges of breast sizes and shapes are similar, allowing the proposed algorithm to be applicable to images from any population. In addition, the use of 3D surface features, such as curvature, eliminates the use of other texture based fiducials, such as nipples, for breast contour detection.

Accurate detection of the IBC contour is important for enabling unbiased and objective measurement of breast aesthetic parameters. The ability to detect the lowest breast contour is very important when determining symmetry between breasts, especially as it relates to how symmetrically the breasts are suspended from the chest wall and how the volume of breast tissue is distributed along the breast curvature (thus helping to make up the overall appearance of the breast contour). The detected IBC contours and the LVP facilitate computation of morphological measures, such as volume, ptosis, and symmetry, which are important for pre-operative planning and post-operative assessment of outcomes in breast reconstruction.
